# Effect of Different Technological Factors on the Gelation of a Low-Lectin Bean Protein Isolate

**DOI:** 10.1007/s11130-022-00956-5

**Published:** 2022-03-05

**Authors:** Helena M. Moreno, M. Teresa Díaz, A. Javier Borderías, Fátima Domínguez-Timón, Alejandro Varela, Clara A. Tovar, Mercedes M. Pedrosa

**Affiliations:** 1 Veterinary Faculty, Department of Food Technology, Avda. Puerta de Hierro, s/n, 28040 Madrid, Spain; 2 Food Technology Department, National Agricultural and Food Research and Technology Institute (INIA), Ctra de La Coruña Km 7.5, 28040 Madrid, Spain; 3 Products Department, Institute of Food Science Technology and Nutrition (ICTAN-CSIC), C/ José Antonio Nováis, 10, 28040 Madrid, Spain; 4 Department of Applied Physics, Faculty of Sciences, University of Vigo, As Lagoas, 32004 Ourense, Spain

**Keywords:** Legumes, White bean protein isolate, Gelation, Mechanical properties, Viscoelasticity

## Abstract

Gelling ability of a bean protein isolate (BPI) obtained from a naturally low-lectin variety (*Phaseolus vulgaris* var. Almonga) was analysed. For that purpose differences on gels processing: concentration (14% and 17%), salt addition (0 and 2%), and pH (6.5 –lot A- and 7 –lot B), were studied to obtain suitable colour, mechanical and viscoelastic properties for making appropriate meat and seafood analogues. Gelation at pH 7 at both 14 and 17% BPI concentrations, produced less rigid, more flexible, time-stable and cohesive gel networks. Colour of the resulting gels was white enough to be considered as an adequate base for making plant-based analogues. The content of total galactoside, inositol phosphates and trypsin inhibitors (bioactive compounds) present in one serving (100 g) of these BPI gels were up to 0.80 mg/g, 8.06 mg/g and 239 TIUs, respectively.

**Supplementary Information:**

The online version contains supplementary material available at 10.1007/s11130-022-00956-5.

## Introduction

The use of plant protein isolates, particularly pulses, is of growing industrial interest due to their functional and technological properties but also their nutraceutical/health beneficial properties [1]. Different scientific studies have associated the consumption of legumes with physiological and health benefits, such as prevention of some types of cancer, cardiovascular diseases, type 2 diabetes, obesity, improvement of the metabolic syndrome, osteoporosis or chronic degenerative diseases [2, 3]. These healthy roles have been linked with pulse proteins and some phytochemicals or bioactive compounds such as galactosides, phytates or phenolic compounds [4].

With the growth of vegetarianism and the demand for non-soy and non-wheat proteins in developed countries, dry beans (*Phaseolus vulgaris* L.) are receiving little by little increased attention for novel food formulations [5]. Although they have similarities, each variety of beans present a unique phytochemical profile [6, 7]. It is important to note that among these phytochemical, the *P. vulgaris* lectin (PHA) is considered as an antinutrient compound since it can be toxic for humans producing vomits, diarrhoea, bloating and interfering with nutrient absorption; however, some potential health benefits (anti-cancer, anti-microbial or reduction diabetes type 2) have been described [2, 6–8] PHA is a glycoprotein highly resistant to thermal denaturation in comparison to other plant proteins, and the presence of this lectin may be the reason for the underutilization of beans as ingredients, mainly, in the elaboration of those food products that are processed at low temperature and/or during a short time. Considering that it is not known the toxicity of all *P. vulgaris* beans they should be considered carefully the lectin intake [8]; therefore, the use of varieties with naturally low-lectin content could be an interesting option for the production of bean protein isolates and for the development of new safe and healthier food products [4, 7, 8]. This is the case of the commercially available common bean var. Almonga used in this study; a white dry bean in the planchada market class, with good culinary quality, and whose consumption has been reported to produce a significant reduction of triglyceride levels [6].

Protein isolates usually are obtained from bean flour by alkaline extraction followed by isoelectric precipitation [4]. Legume proteins comprise water-soluble albumins, globulins (legumins and vicilins) soluble in salt solutions, the family of lectin-related proteins, and a minor proportion of prolamins and glutelins soluble in dilute acid/base solutions. Thus, the final composition of the BPI plays an important role on the techno-functional properties, which are of great importance for gelation ability [9]. Moreover, there are some factors such as pH, ionic strength and protein concentration that also affect gel formation [10]. Taking that into account, mechanical properties are important for the behaviour of food materials, and especially of gels, which are major features in consumer perception and acceptance [11]. These factors will result in gels with different characteristics such as texture, viscoelasticity, colour and flavour as the basis for processing final products that mimic seafood or meat products [12].

One important parameter to take under consideration during BPI gelation is the effect of pH on proteins and their influence on techno-functional properties. It has suggested that *β*-sheet can be reduced by acidic pH, thus reducing the stability of the network structure. Also, NaCl may increase the presence of disordered structures such as random coil by reducing *β*-sheet [1], thus altering the physicochemical properties of the resulting gel.

The aim of this paper was to throw light on the gelation ability of a bean protein isolate from a variety with a low-lectin content (*P. vulgaris var.* Almonga). The effects of BPI concentration, presence of salt, and pH were studied with a view to developing gels with suitable textures for making meat and seafood plant-based analogues, as well as with a bioactive compounds content sufficient to exert an effective health protective effect. To our knowledge this is the first research work done on the gelation ability of this low-lectin bean variety based on the electrophoretic analysis, mechanical and viscoelastic properties.

## Materials and Methods

The methods used to determine the proximate composition of the bean protein isolate and their techno-functional properties (pH and last gelation capacity), as well as the mechanical properties (puncture test), the electrophoretic profile, colour, viscoelasticity (oscillatory tests) of the gels and their bioactive compounds content are described in the supplementary material and methods file (Electronic supplementary material-1).

### Preparation of Bean Protein Isolates (BPI)

Beans (*P. vulgaris* var. Almonga -Benjamín Rodríguez Álvarez, León, Spain-) was used to prepare the bean protein isolate by alkaline extraction followed by isoelectric precipitation (bean composition and additional details are given in Electronic supplementary material −1).

### Preparation of Bean Protein Isolate Gels

Samples were prepared using the obtained BPI at three concentrations (14, 17 and 20%). The lowest BPI concentration corresponds to the LGC value determined previously (Online resource 1) and that secure a suitable gel for the purpose of this work. The addition of 2% of NaCl and the pH adjustment of the gels resulted into the different sample codes: G14-A, G17-A and G-20-A with a final of pH 6.5 and G14-B, G17-B, G20-B with a final of pH 7.0 for 0% NaCl, and 2G14-A, 2G17-A, 2G20-A, 2G14-B, 2G17-B, 2G20-B for the samples containing 2% NaCl (additional details are given in Electronic supplementary material −1).

### Puncture Test

It was firstly performed on all the gels prepared in order to select those with the most suitable mechanical properties (Online resource 1). The selected samples were included in the subsequent analyses and the others were discarded.

### Dynamic Rheometry (Stress Sweep Tests and Mechanical Spectra) of Bean Protein Isolate Gels

Small amplitude oscillatory shear (SAOS) tests were performed for Lots A and B of BPI gels with the better mechanical properties from puncture tests (14 and 17% BPI concentration and 0% NaCl) (additional details are given in Electronic supplementary material −1).

### Statistical Analysis

One-factor ANOVA analysis was carried out with the SPSS® computer programme (SPSS Inc., Chicago, IL, USA) and average differences were evaluated by the Tukey test using a 95% confidence interval. Viscoelastic data were tested with expanded uncertainty limits as the maximum and minimum deviation from the respective mean values. Trends were considered significant when means of compared sets differed by* p* < 0.05 (Student’s test).

## Results and Discussion

### Proximate Composition and Techno-Functional Properties of Bean Protein Isolate

The chemical composition can influence the techno-functional properties of the isolate; in particular thermally induced gelation behaviour [9]. BPI moisture and ash content was 1.08% ± 0.01 and 4.70 ± 0.06, respectively. The content of total protein (75.42% ± 0.91 d.w.) and fat (3.86% ± 0.20 d.w.) of the BPI was in the range 70–77% and 2.5–4%, respectively, reported in the literature, and the proportion of carbohydrates was 16.03% ± 0.77 (d.w.), of which 0.73% ±0.07 (d.w) was starch and 3.27% ± 0.07 (d.w.) was dietary fibre. This proximate composition was similar to that reported by other authors for different varieties of kidney bean protein isolates [13]. The high proportion of soluble protein (44.1% ± 1.5 d.w.) present in the BPI indicated that this isolate is suitable for use as an ingredient in different food applications. Vicilins and legumins (the main storage legume proteins) can form thermally-induced gels, although their gelation behaviour is slightly different and changes in the vicilin/legumin can affect the functionality of the protein isolate [14]. The legumin/vicilin ratio was 15.25, indicating that this BPI is a legumin-rich isolate. The pH of the BPI powder in water dispersion was 5.33 ± 0.02. The legume protein isolates exhibit better techno-functional properties (such as gelation, foaming or emulsifying properties) at pH values close to neutral [15], for that reason the pH of the samples was raised up to 6.5 (lot A) and 7.0 (lot B). LGC was 14% BPI at natural pH (5.33), so, 14, 17 and 20% BPI gels were checked at both pHs to determine their mechanical characteristics according to the aim of this work. This LGC value of BPI was similar to that reported by some authors for red kidney bean faba bean or chickpea isolates [12, 15–17], and lower than that reported for pea or soybean isolates, these differences can be related to the influence of the type and variety of pulse studied [13].

### Mechanical Properties: Puncture Test on Bean Protein Isolate Gels

**Fig. 1:**
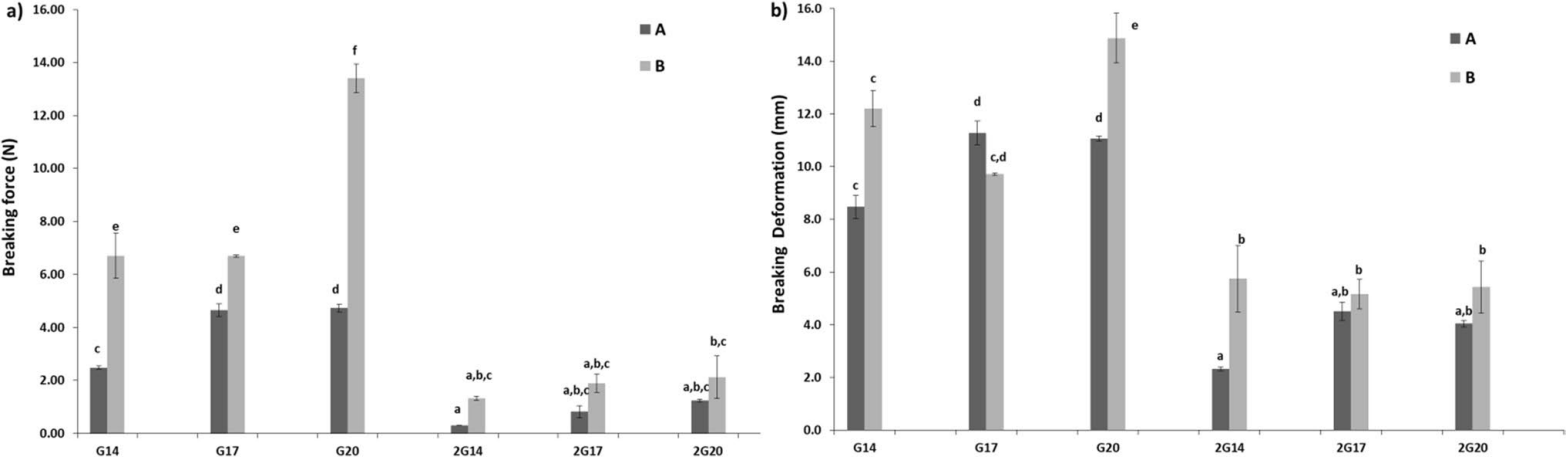
Breaking force (**a**) and breaking deformation (**b**) for bean protein isolate (BPI) gels at different pHs. Different capital letters for each value indicate significant differences *p* < 0.05 among samples of pH = 6.5 (A –dark grey-) and pH = 7 (B –light grey-) without salt. Different lowercase letters for each value indicate significant differences *p* < 0.05 among samples of pH A and B with 2% NaCl

The effect of salt (0 and 2% NaCl) on the mechanical properties of the gels were tested through a puncture test. The addition of 2% salt dramatically decreased the breaking force (BF) and breaking deformation (BD) for 2G14-A, 2G14-B, 2G17-A, 2G17-B, 2G20-A, 2G20-B vs G14-A, G14-B, G17-A, G17-B, G20-A, G20-B (Fig. 1a and b). This is probably due to the presence of saline ions that alter the electrostatic balance of the charges that stabilized the BPI gel network without salt. This result indicates that higher ionic strength at pH higher than that of the isoelectric point increased the repulsive electrostatic forces among proteins weakening the inter-protein packing and consequently the mechanical resistance to gel rupture was reduced. The effect of NaCl concentration has also been related to the protein content in the medium, so the higher the ionic strength, the higher is the protein concentration required to form a proper gel network [18, 19]. This is consistent with the results shown since the increase in BF and BD associated to the greater BPI concentration, was mitigated in presence of salt. Besides that, all gels made with 2% of NaCl (2G14-A, 2G14-B, 2G17-A, 2G17-B, 2G20-A, 2G20-B) had poor mechanical properties to be considered as proper gels to the aim of this study and were discarded for further analysis.

Focusing on the pH of gels, the narrow difference between them (6.5 vs 7.0) had a considerable impact on the gel properties, as evidenced by the higher values of BF (*p* < 0.05) in gels B (pH 7.0) vs A (pH 6.5), irrespective of BPI concentration (Fig. 1a). This could be related to the prevalent surface charge of the proteins at each pH [19]. Protein unfolding and aggregation is required to form an adequate gel, and it has been reported that legume protein isolates exhibit better functional properties at pH values close to neutral [14, 15]. Gels containing 20% BPI without salt (G20-B) were also rejected for the purpose of the study due to the extremely high values of BF and BD which are far from those desired to make meat and seafood products analogues.

### Electrophoretic Profile

Bean proteins are composed mainly of vicilin (7S) and legumin (11S) and in the BPI of this low-lectin variety, vicilins made up 51.47 ± 0.98% of the total proteins and legumins 72.1 ± 1.0%. Vicilin is a kind of glycoprotein frequently non-covalently associated in trimers (7–8S globulins) with a molecular weight of 150–250 kDa, or even hexamers, while the legumin consists of acidic (about 40 kDa) and basic (20 kDa) subunits linked by disulphide bonds [20].

**Fig. 2:**
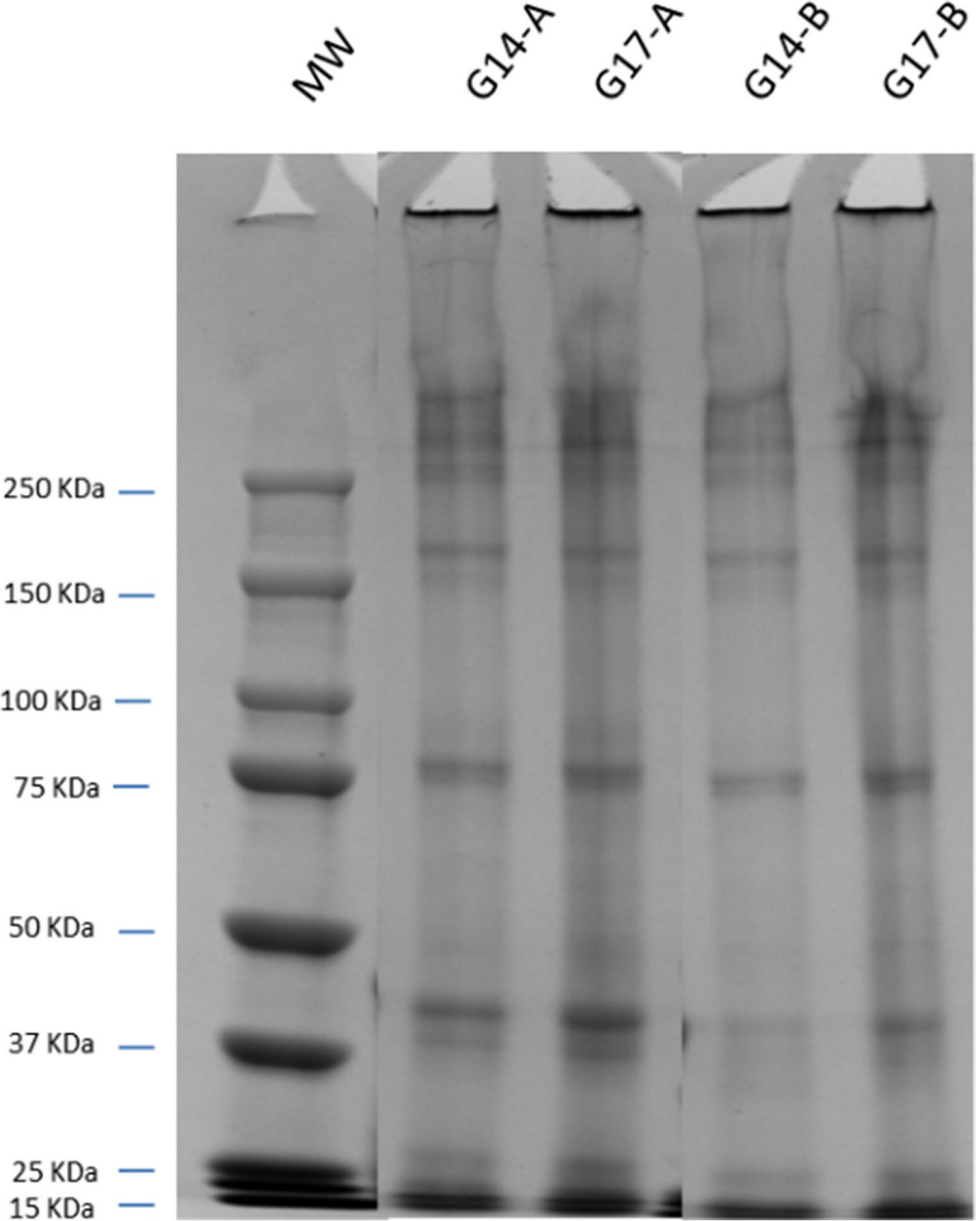
Electrophoretic profile for bean protein isolate (BPI) gels at different pHs

The electrophoretic profiles of the above selected gels according to their mechanical properties (G14-A, G14-B, G17-A and G17-B) were similar, with bands corresponding to a molecular weight around 250 and 160 kDa (vicilins) and 75, 40, 25 and 15 kDa (Fig. 2), which are assumed to be legumin subunits [21]. As reported earlier, this low-lectin BPI is legumin-rich, which would explain the lower intensity of the vicilin bands as compared to legumins, particularly in the samples with 17% BPI (Lot A and B).

The presence of noticeable aggregates retained in the stacking electrophoresis gel [22] seems to be due to MTGase activity, which has the ability to form intermolecular aggregates [23] and in the formation of dimers and polymers of 11S and 7S [22, 23].

Comparison of the A and B groups of gels showed that the bands most affected, regardless of BPI concentration, are the ones of the lowest molecular weight (40 kDa and below), whose intensity is lower in B gels. The reason for the reduction in intensity of these bands (G14-B and G17-B) could be that the location of the reactive residues to MTGase in these proteins is more accessible [24].

### Colour of Bean Protein Isolate Gels

Colour parameters (L*, a* and b*) and whiteness index (WI) of BPI gels are presented in Table 1. L* varied between 73.55 ± 0.15 and 75.78 ± 0.47, indicating high luminosity; a* ranged from 0.83 ± 0.21 to 0.04 ± 0.03, meaning no tendency to redness; while all the gels showed a positive b* ranging from 12.95 ± 1.03 to 15.25 ± 0.04, indicating a trend to yellowness, especially in the samples with higher BPI content regardless of the pH (G17-A and G17-B). Calculation of WI revealed a significantly lower “perceived” whiteness in samples with the highest BPI content regardless of pH (G17-A and G17-B) as in the case of b*. This could be due to the yellowness/creamy colour of that particular BPI. All these values indicated that the gels were very light in colour and thus suitable for making different food analogues in which light colours are desired or in which the colour can be easily modified.

**Table 1:** Colour values of the different bean protein gels

Samples	L*	a*	b*	Whiteness Index
G14-A	75.78 ± 0.47^c^	−0.21 ± 0.33^b^	13.92 ± 0.39^a,b^	34.02 ± 1.64^c^
G17-A	74.80 ± 0.45^b^	0.04 ± 0.03^b^	14.80 ± 0.19^b,c^	30.41 ± 0.20^b^
G14-B	73.55 ± 0.15^a^	−0.83 ± 0.21^a^	12.95 ± 1.03^a^	34.69 ± 2.19^c^
G17-B	74.09 ± 0.25^a,b^	−0.01 ± 0.04^b^	15.25 ± 0.04^c^	28.35 ± 0.28^a^

Values are given as mean ± standard deviation. a-c: Different superscript letters for each parameter indicate significant differences (*p* < 0.05) among samples in each parameter

### Oscillatory Measurements

#### Stress Sweeps

The limit values of the linear viscoelastic (LVE) range: the stress amplitude (*σ*_*max*_), and the strain amplitude (*γ*_*max*_) (Table 2), evidence that BPI gels had considerable structural stability and adequate conformational flexibility [12].

**Table 2:** Effect of pH and BPI concentration on the viscoelastic parameters from small amplitude oscillatory shear (SAOS) tests for bean protein isolate (BPI) gels at 20 °C. 2a Viscoelastic parameters for the linear viscoelastic (LVE) range at 1 Hz

Sample	σ_max_ (Pa)	γ_máx._ (%)	G* (kPa)
G14-A	1500 ± 150^a^	4.23 ± 0.71^a^	39.0 ± 3.8^c^
G14-B	1989 ± 198^b^	10.9 ± 1.1^c^	18.3 ± 1.9^a^
G17-A	3796 ± 380^d^	6.76 ± 0.29^b^	58.6 ± 2.6^d^
G17-B	3000 ± 300^c^	9.32 ± 0.62^c^	32.2 ± 2.2^b^

Values are given as mean ± expanded uncertainty limit (EUL). a-d: Different superscript letters for each parameter indicate significant differences *p* < 0.05 among samples

At lower BPI concentration, both *σ*_*max*_ and *γ*_*max*_ increased significantly (*p* < 0.05) with increasing pH; specifically *σ*_*max*_ increased by 33% and *γ*_*max*_ increased by 158% in G14-B vs G14-A (Table 2). This result indicates that the slight increase in pH noticeably enhanced the structural stability and more the conformational flexibility. Moreover, there was a significant decrease (53%) in gel strength (G*) between G14-A and G14-B gels (Table 2), indicating a considerable reduction in the overall rigidity of the protein network at pH = 7. A possible explanation for these findings is that beyond the isoelectric range -pH =4.8 and 5.5- of bean proteins [25], a fine-stranded structure would be formed and hence the diameter of the protein strands would decrease [26], which in turn would reduce the overall (elastic and viscous) resistance to deformation (low G*). In the case of 17% BPI, the analogous pH increase was reflected in a similar qualitative trend in the viscoelastic parameters (*γ*_*max*_ and G*), although with lower percentages than at 14% BPI concentration (Table 2). Naturally, at the higher protein concentration a more compact protein matrix was formed which partially screened the structural effect of pH on the gel-network density. So, a more fine-stranded structure (Gels-B) would favour a more accessible location of the reactive residues observed in the electrophoretic profiles.

At fixed pH, the increase in BPI concentration, naturally increased G* due to the greater network density in both G17-A vs G14-A and G17-B vs G14-B. However, it should be noted that in lot A gels, *γ*_*max*_ was significantly lower at the lower BPI concentration (G14-A vs G17-A). This is a peculiar characteristic of globular proteins, since at lower concentrations the protein aggregates contributing to the structure are shorter and more compact [27] than at higher protein concentrations. That is why a less deformable gel network was formed for G14-A vs G17-A (Table 2).

#### Frequency Sweeps

**Fig. 3:**
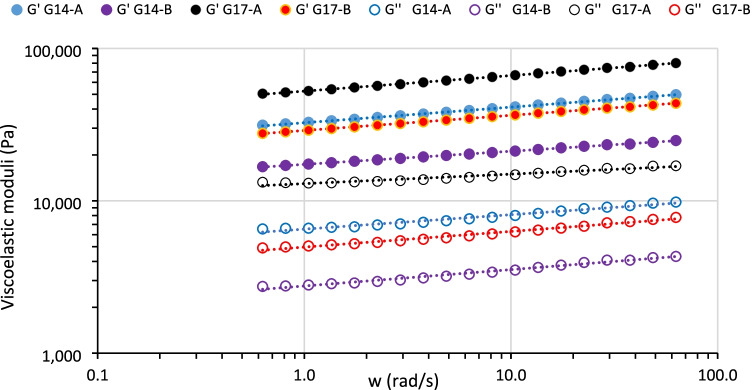
Mechanical spectra of gels at 14% and 17% BPI concentration for lots A and B at 20 °C

Mechanical spectra of the BPI gels with 14 and 17% BPI concentration indicated that samples were true gels at both concentrations because G’ > G” [28] with slight frequency dependence in both G’ and G” (Fig. 3). To quantify their frequency dependence both parameters were fitted to the power law (Eqs. (1) and (2)):

1 $${G}^{\prime }={G}_0^{\prime}\cdot {\upomega}^{n^{\prime }}$$

2 $${G}^{\prime \prime }={G}_0^{\prime \prime}\cdot {\upomega}^{n^{\prime \prime }}$$

where *G*_*0*_*’* and *G*_*0*_*”* are the elastic and viscous moduli at 1 rad/s, and *n’* and *n”* denote the rate of increase in *G’* and *G”* respectively with increasing the angular frequency (ω). The loss factor $\mathit{\tan}\updelta =\frac{G_0^{\prime \prime }}{G_0^{\prime }}$ is a measurement of the solid-like character of gels and provides useful information about the energy of interactions in the gel network [29].

At fixed BPI concentration, the increase in pH reduced both *G*_*0*_*’* and *G*_*0*_*”* parameters irrespective of the concentration (Table 3). Thus, at pH = 7, outside the pI range of bean proteins, the electrostatic charge in the amino acid residues would increase, so the inter-chain repulsive forces augmented and consequently a more hydrated protein network was formed which would explain the observed softening of the solid matrix in gels B vs A at both 14 and 17% BPI concentrations (Table 3). This fact is consistent with the lower L* values for lot B vs A, since L* shows the light scattered by protein aggregates in the gel network. Therefore, at higher pH the BPI gel network had a less compact protein-matrix, reducing the effect of diffuse reflection and consequently a decrease of L* [30]. In addition, *tanδ* values were significantly lower (*p* < 0.05) in gels B vs A (Table 3) indicating that at pH = 7 the ideal network-fraction was enhanced irrespective of BPI concentration [31]. This fact is equivalent to a greater lifetime of protein-protein interactions than that for gels at pH = 6.5 [30].

**Table 3:** Effect of pH and BPI concentration on the viscoelastic parameters from small amplitude oscillatory shear (SAOS) tests for bean protein isolate (BPI) gels at 20 °C. 2b Fit parameters of mechanical spectra (Eqs. 1 and 2)

Samples	G_o_’ (kPa)	n’	r^2^	G_o_” (kPa)	n”	r^2^	tan δ
G14-A	32.6 ± 3.2^b^	0.102 ± 0.001^b^	0.999	6.5 ± 1.1^b^	0.095 ± 0.004^c^	0.977	0.199 ± 0.002^c^
G14-B	17.35 ± 0.73^a^	0.086 ± 0.001^a^	0.999	2.76 ± 0.53^a^	0.107 ± 0.003^b^	0.984	0.166 ± 0.003^a^
G17-A	52.5 ± 2.6^c^	0.102 ± 0.001^b^	0.999	13.0 ± 3.7^c^	0.063 ± 0.003^a^	0.953	0.227 ± 0.006^d^
G17-B	29.0 ± 2.1^b^	0.100 ± 0.001^b^	0.999	4.97 ± 0.53^b^	0.103 ± 0.003^b^	0.986	0.172 ± 0.002^b^

Values are given as mean ± expanded uncertainty limit (EUL). a-d Different letters for each parameter indicate significant differences *p* < 0.05 among samples

At fixed pH, the increase in BPI concentration logically increased both *G*_*0*_*’* and *G*_*0*_*”*, indicating a denser protein matrix in a more packed network. However, *tanδ* significantly increased (p < 0.05) in G17 vs G14 irrespective of pH (Table 3). This trend indicates that higher (17% BPI concentration, produced a more rigid and less energy-stable inter-protein bonds resulting in a less ideal network-fraction at 17% vs 14% BPI [12] more evident in lot A samples. This result is consistent with the greater difference between the two exponents (*n’ > n”)* in G17-A vs G14-A (Table 3), indicating that at the higher BPI concentration, a less time-stable protein matrix was formed, as evidenced by the faster loss of the ideal network fraction (high *n’*) compared to the non-ideal part (*n”*). In lot B the same increase in BPI concentration produced similar values for *n’* (0.100 ± 0.001) and *n”* (0.103 ± 0.003) at 17% BPI concentration (Table 3). These low and comparable exponents (*n’* and *n”*) corroborate the greater time stability of the protein network [30] produced by the higher BPI concentration at pH = 7 (G17-B).

### Bioactive Compounds of Selected Bean Protein Isolate Gels

The content of some bioactive compounds present in different foods is affected by the food matrix and the manufacturing process, increasing or decreasing their content in the end-product. Therefore, it is recommended to determine their presence in novel foods such as vegetable meat or seafood analogue gels.

The studied BPI was analysed in a previous work [2] and it was characterized by the presence of inositol phosphates (32.67 mg/g), *α*-galactosides (14.27 mg/g), lectins (0.73% PHA or 0.60 HU/mg) and trypsin inhibitors (24.17 TIU/mg). Stachyose and phytic acid, respectively, were the main *α*-galactoside and inositol phosphates found in the isolate. Regarding the PHA content, notably, the BPI contained a very low lectin content since the Almonga variety is a low-lectin seed [5] and thus this BPI can be used safely in the development of new products, especially made at low temperature and/or short time. The higher percentage of BPI produced the larger amount of bioactive compounds in the vegetable gels (Table 4). The content of bioactive compounds in these gels is higher of that reported by Borderias et al. [30] in pea-surimi-like gels containing up to 15% of pea protein isolate (PPI). This is due to the BPI presented higher amount of bioactive compounds that the commercial PPI studied by these authors. In general, the concentration of these compounds determined in the gels was low; however, the healthy effect of these compounds depends on their intake, and there is not always a threshold level recommended to achieve the healthy effect [2]. Trypsin inhibitors are known to be anticarcinogenic compounds; it has been reported that the traditional Japanese diet contains around 420 protease inhibitor units *per* day, and a consumption of 25–800 units per day during three month exerts a protective effect against cancer in humans [2] (and citations therein).

**Table 4:** Bioactive compounds content (wet basis) on gels elaborated at two BPI concentrations (14 and 17%)

Bioactive compound	G 14%	G 17%
Inositol phosphates (IP) (mg/g)		
IP3	0.06 ± 0.00^b^	0.07 ± 0.00^b^
IP4	0.23 ± 0.00^c^	0.44 ± 0.03^b^
IP5	0.97 ± 0.03^c^	1.96 ± 0.05^b^
IP6	2.55 ± 0.10^c^	6.08 ± 0.04^b^
Total IP	3.79 ± 0.08^c^	8.62 ± 0.12^b^
Trypsin inhibitors (TIU/mg)	1.42 ± 0.01^c^	2.39 ± 0.02^b^
Lectins (%PHA)	n.d.	n.d.
*α*-galactosides (mg/g)		
Raffinose	3.01 ± 0.11^b^	4.08 ± 0.07^a^
Ciceritol	0.81 ± 0.07^b^	1.01 ± 0.01^b^
Stachyose	2.77 ± 0.14^c^	3.90 ± 0.10^b^
Total galactosides	5.78 ± 0.15^c^	7.98 ± 0.13^b^

Values are given as mean ± standard deviation; n = 4; n.d. = not detected

According to Martínez-Villaluenga et al. [32] consumption of 3 g/day of *α*-galactosides produced a clear prebiotic effect, increasing the growth of beneficial microbiota, and improving the immune system. Hurrell et al. [33] reported a significant increase in iron absorption when IP6 was below 10 mg/g protein in one serving of the meal. Most of the surimi manufacturer recommended in the product label consuming one serving/day of around 70-125 g. Thus, taking these gels as vegetable surimi-like gels, one serving of 100 g was chosen to determine the content of the bioactive compounds in one meal. Thus, one serving of these BPI gels supplied from 0.58 g (G14) to 0.80 g (G17) of total galactosides and one serving of G14 and G17 contained 3.38 and 8.06 mg of IP6 *per* g meal protein. In addition, taking into account the presence of these phytochemicals in the vegetable gels, the BPI may be considered an added-value ingredient in the development of new foods with specific health roles. One serving of G14 and G17 samples provided 142 and 239 TIUs of trypsin inhibitors, amounts that are in the effective range for prevention or suppression of carcinogen-induced effects [2, 4]. The amount of bioactive compounds supplied by one serving of these BPI gels are higher of that reported that reported by Borderias et al. [30] in pea-surimi-like gels. According to some authors [33, 34] consumption of food with total galactosides content lower than 12 mg/g and total inositol phosphates <20 mg/g (like the studied gels) produced low flatulence and afforded a mineral availability similar to commercial formulas based on soybean protein.

## Conclusions

From the results of the physicochemical, mechanical and viscoelastic properties of the resulting gels it can be concluded that the low-lectin BPI is a legumin-rich isolate with great gelation ability at pH 7 (Lot B) in 14 and 17% BPI concentrations. Rheologically, they rendered less rigid gel networks with improved conformational flexibility, cohesiveness, and time stability than the lot A obtained al pH 6.5. These findings permit to deduce that Lot B (pH 7) made with 14 and 17% BPI with 5 U/g of MTGase was the most suitable for the study objective. Regarding the colour, all the gels showed a slight yellowness/creamy that easily permit gel colour modification. In addition, the light colour of the gels in combination with the viscoelastic and mechanical properties of Lot B, regardless of BPI concentration, would allow the formation of gels with a wide range of textures as the basis of making meat-products and seafood analogues from a vegetal raw material. From a health point of view and according to the literature gels of Lot B contain enough bioactive content to exert a protective effect against some carcinogenic processes, to improve iron absorption, and to produce a prebiotic effect on the microbiota without the discomfort of flatulence.

## Supplementary Information

ESM 1 (DOCX 35 kb)

## Data Availability

Authors declare that datasets are available on request. The raw data supporting the conclusions of this article will be made available by the authors, without undue reservation.

## References

[1] Shevkani K, Singh N, Chen Y, Kaur A, Yu L (2019). Pulse proteins: secondary structure, functionality and applications. J Food Sci Technol.

[2] Muzquiz M, Varela A, Burbano C, Cuadrado C, Guillamon E, Pedrosa MM (2012). Bioactive compounds in legumes: pronutritive and antinutritive actions. Implications for nutrition and health. Phytochem Rev.

[3] Carbonaro M (2021) Nutraceutical perspectives of pulses. In: Tiwari BK, Gowen A, McKenna B (eds) Pulse Foods, Academic Press, San Diego, Chapter 17. pp. 423–460. 10.1016/B978-0-12-818184-3.00017-9

[4] Pedrosa MM, Varela A, Domínguez-Timón F, Tovar CA, Moreno HM, Borderías AJ, Díaz MT (2020). Comparison of bioactive compounds content and techno-functional properties of pea and bean flours and their protein isolates. Plant Foods Hum Nutr.

[5] Gupta S, Chhabra GS, Liu C, Bakshi JS, Sathe SK (2018). Functional properties of select dry bean seeds and flours. J Food Sci.

[6] Olmedilla-Alonso B, Pedrosa M, Cuadrado C, Brito M, Asensio-S-Manzanera C, Asensio-Vegas C (2013). Composition of two Spanish common dry beans (*Phaseolus vulgaris*), 'Almonga' and 'Curruquilla', and their postprandial effect in type 2 diabetics. J Sci Food Agric.

[7] Pedrosa MM, Cuadrado C, Burbano C, Muzquiz M, Cabellos B, Olmedilla-Alonso B, Asensio-Vegas C (2015). Effects of industrial canning on the proximate composition, bioactive compounds contents and nutritional profile of two Spanish common dry beans (*Phaseolus vulgaris* L.). Food Chem.

[8] He S, Simpson BK, Sun H, Ngadi MO, Ma Y, Huang T (2018). Phaseolus vulgaris lectins: a systematic review of characteristics and health implications. Crit Rev Food Sci Nutr.

[9] Kiosseoglou V, Paraskevopoulou A, Tiwari BK, Gowen A, McKenna B (2011). Functional and physicochemical properties of pulse proteins. Pulse Foods.

[10] Makri E (2005). Study of functional properites of seed storage proteins from indigenous Europan legume crops (lupin, pea, broad bean) in admixture with poysaccharides. Food Hydrocoll.

[11] Szczesniak AS (2002) Texture is a sensory property. Food Qual Pref 13:215–225. 10.1016/s0950-3293(01)00039-8

[12] Moreno HM, Domínguez-Timón F, Díaz MT, Pedrosa MM, Borderías AJ, Tovar CA (2020). Evaluation of gels made with different commercial pea protein isolate: rheological, structural and functional properties. Food Hydrocoll.

[13] Tang C-H (2008). Thermal denaturation and gelation of vicilin-rich protein isolates from three Phaseolus legumes: a comparative study. LWT-Food Sci Technol.

[14] Barac M, Pešić M, Stanojevic S, Kostić A, Cabrilo S (2015). Techno-functional properties of pea (*Pisum sativum*) protein isolates: a review. Acta Per Technol.

[15] Adebiyi AP, Aluko RE (2011). Functional properties of protein fractions obtained from commercial yellow field pea (*Pisum sativum* L.) seed protein isolate. Food Chem.

[16] Fernández-Quintela A, Macarulla MT, del Barrio AS, Martínez JA (1997). Composition and functional properties of protein isolates obtained from commercial legumes grown in northern Spain. Plant Foods Hum Nutr.

[17] Hayat I, Ahmad A, Masud T, Ahmed A, Bashir S (2013) Nutritional and health perspectives of beans (*Phaseolus vulgaris* L.): an overview. Crit Rev Food Sci Nutr 54:580–592. 10.1080/10408398.2011.596639 10.1080/10408398.2011.596639 24261533

[18] Akintayo ET, Oshodi AA, Esuoso KO (1999) Effects of NaCl, ionic strength and pH on the foaming and gelation of pigeon pea (*Cajanus cajan*) protein concentrates. Food Chem 66:51–56. 10.1016/S0308-8146(98)00155-1

[19] Lawal OS (2004) Functionality of African locust bean (*Parkia biglobossa*) protein isolate: effects of pH, ionic strength and various protein concentrations. Food Chem 86:345–355. 10.1016/j.foodchem.2003.09.036

[20] Meng GT, Ma C-Y (2001) Thermal properties of *Phaseolus angularis* (red bean) globulin. Food Chem 73:453–460. 10.1016/S0308-8146(00)00329-0

[21] Mühling M, Gilroy J, Croy RRD (1997) Legumin proteins from seeds of *Phaseolus vulgaris* L. J Plant Physiol 150:489–492. 10.1016/S0176-1617(97)80103-4

[22] Liu C, Damodaran S, Heinonen M (2018). Effects of microbial transglutaminase treatment on physiochemical properties and emulsifying functionality of faba bean protein isolate. LWT-Food Sci Technol.

[23] Damodaran S, Agyare KK (2013). Effect of microbial transglutaminase treatment on thermal stability and pH-solubility of heat-shocked whey protein isolate. Food Hydrocoll.

[24] Djoullah A, Djemaoune Y, Husson F, Saurel R (2015). Native-state pea albumin and globulin behavior upon transglutaminase treatment. Process Biochem.

[25] Sathe SK (2002). Dry bean protein functionality. Crit Rev Biotechnol.

[26] Munialo CD, van der Linden E, de Jongh HHJ (2014). The ability to store energy in pea protein gels is set by network dimensions smaller than 50nm. Food Res Int.

[27] Zhang Y-H, Wen Q-B, Yang X-Q, Li L, Deng W-L (2010) Thermal aggregation and gelation of kidney bean (*Phaseolus vulgaris* L.) protein isolate at pH 2.0: influence of ionic strength. Food Hydrocoll 24:266–274. 10.1016/j.foodhyd.2009.10.002

[28] Cando D, Moreno HM, Tovar CA, Herranz B, Borderías AJ (2014). Effect of high pressure and/or temperature over gelation of isolated hake myofibrils. Food Bioprocess Technol.

[29] Herranz B, Borderías AJ, Solas M, Tovar CA (2012). Influence of measurement temperature on the rheological and microstructural properties of glucomannan gels with different thermal histories. Food Res Int.

[30] Borderías AJ, Tovar CA, Domínguez-Timón F, Díaz MT, Pedrosa MM, Moreno HM (2020). Characterization of healthier mixed surimi gels obtained through partial substitution of myofibrillar proteins by pea protein isolates. Food Hydrocoll.

[31] Nijenhuis KT (1997) In: Nijenhuis KT(ed) Thermoreversible networks. Viscoelastic properties and structure of gels. Advances in Polymer Science. Springer-Verlag Berlin Heidelberg. 10.1007/BFb0008699

[32] Martınez-Villaluenga C, Torres A, Frias J, Vidal-Valverde C (2010). Semolina supplementation with processed lupin and pigeon pea flours improve protein quality of pasta. LWT-Food Sci Technol.

[33] Hurrell RF, Juillerat MA, Reddy MB, Lynch SR, Dassenko SA, Cook JD (1992). Soy protein, phytate, and iron absorption in humans. Am J Clin Nutr.

[34] Fredrikson M, Alminger ML, Carlsson N-G, Sandberg A-S (2001). Phytate content and phytate degradation by endogenous phytase in pea (*Pisum sativum*). J Sci Food Agric.

