# Supplementary material for: Effect of Different Technological Factors on the Gelation of a Low-Lectin Bean Protein Isolate
Source: Plant Foods Hum Nutr. 2022 Mar 5;77(1):141–9. doi: 10.1007/s11130-022-00956-5 (PMC8993736; doi:10.1007/s11130-022-00956-5)
Supplement: Supplementary file 1 — (DOCX 35 kb) [file 11130_2022_956_MOESM1_ESM.docx]

**Supplementary Material 1 Moreno et al.**

**Materials and methods**

**Preparation of bean protein isolates (BPI)**

Beans (*Phaseolus vulgaris* var. Almonga, (Benjamín Rodríguez Álvarez, León, Spain) was used to obtain their protein isolate. The composition of beans was 10.63±0.98% moisture, 25.16±0.32% protein, 10.08±0.91% soluble protein, 2.51±0.16% fat, 4.81±0.04% ash, 67.53±0.99% total carbohydrate, 26.27±0.60% starch, and 21.6±1.9 % fibre. The BPI was obtained by alkaline extraction followed by isoelectric precipitation according to the method of Pedrosa et al. [1]. Briefly, the bean flour was dispersed in distilled water (1:6 w/v), adjusted to pH 9 with 0.1 N NaOH, and stirred continuously for 1 h at room temperature, followed by centrifugation at 9000 rpm for 20 min at 5 °C. This procedure was repeated one more time on the residue. Supernatants were pooled, adjusted to pH 4.30 using 1 N HCl, and stirred continuously for 2 h at 4 °C, followed by decantation overnight at 4 °C. The precipitate formed was recovered by centrifugation at 9000 rpm for 20 min at 5 °C; then, the pellet was suspended in distilled water, adjusted to pH 6 using 1N NaOH, and freeze dried to produce the BPI.

**Proximate composition and techno-functional properties of the bean protein isolate**

Total protein, legumin and vicilin content was analysed with a LECO® CNS-2000 (N x 5.45) and soluble protein by the Bradford method [2]. Legumin and vicilin fractionation was carried out by isoelectric precipitation and fractionation based on differential protein solubility according to Cuadrado et al [3]. The pH was measured according to the official AOAC procedure [4]. The least gelation concentration (LGC) of BPI was determined by the method of Chau & Cheung [5] as the lowest BPI concentration that did not fall or slip after inversion of the test tube in order to know the BPI concentration needed for the formation of a gel, with enough textural characteristics to simulate a muscle.

**Preparation of bean protein isolate gels**

Samples were prepared using 14%, 17% and 20% BPI from *Phaseolus vulgaris* var. Almonga. The lowest BPI concentration used corresponds to the LGC value determined previously and that secure a suitable gel for the purpose of this work. The influence of 2% of NaCl on BPI gelation was analysed. This salt amount was selected based on previous works on pea protein gelling, in which the presence of salt increased the gel strength [6,7]. Two different phosphate buffers were used instead of water to dissolve BPI, which is *per se* relatively low (5.33±0.02) obtaining a final pH in both concentrations of 6.5 (A) and 7.0 (B). According to the studied parameters, samples were coded as collected in table 1.

Table 1: Samples´ code.

| **Samples** | **Final pH** | **NaCl (%)** |
| --- | --- | --- |
| G14-A | 6.5 | 0.0 |
| G17-A |  |  |
| G20-A |  |  |
| 2G14-A |  | 2.0 |
| 2G17-A |  |  |
| 2G20-A |  |  |
| G14-B | 7.0 | 0.0 |
| G17-B |  |  |
| G20-B |  |  |
| 2G14-B |  | 2.0 |
| 2G17-B |  |  |
| 2G20-B |  |  |

Lastly, the mixture was homogenized at 80 °C/5 min. Then, five units per gram of protein of microbial transglutaminase WM (MTGase) (99% Maltodextrine and 1% enzyme with an activity approx. 100 U/g of powder; Ajinomoto Co., North America, Inc., USA) was added to each sample from lots A and B after reducing the temperature to 40 °C. MTGase addition is related to the researchers´ previous analyses that indicated a very poor texture of the gels, especially in deformation, if MTGase is not added.

Samples were finally gently homogenized under vacuum for 2 minutes (Mestra, Iris 2, Bilbao, Spain) in all cases in order to extract the air. Resulting samples were stuffed into 35 mm Krehalon casings (Amcor group Flexibles Hispania S.L., Barcelona, Spain) and subjected to a setting/heating treatment (40 °C/1 hour + 5 °C / overnight + 90 °C/30 min). Samples were cooled down to 5 °C and kept at that temperature till the analyses were carried out.

**Mechanical properties: Puncture test on bean protein isolate gels**

Puncture test was carried out at room temperature (25 °C) on lots A and B of BPI gels at several BPI concentrations (14%, 17% and 20%) with and without NaCl. Puncturing was performed on gels of diameter 35.0 mm and height 30.0 mm using a 5 mm diameter rounded ended cylindrical stainless steel plunger attached to a 50 N cell connected to the crosshead on a TA-XT plus Texture Analyser (Texture Technologies Corp., Scarsdale, NY, USA). Breaking force (BF) and breaking deformation (BD) were determined from force-distance curves derived at 1.0 mm s^-1^ crosshead speed. Measurements were carried out in triplicate. This analysis was firstly performed on all the gels in order to select those with the best mechanical properties. The selected samples were included in the subsequent analyses and the others were discarded.

**Electrophoretic profile**

The SDS-PAGE electrophoresis profiles of bean protein isolate gels selected by puncture test from lots A and B, with 14% and 17% BPI and 0% NaCl (G14-A, G17-A, G14-B, G17-B) were analysed. To that end, samples were dissolved in a SDS-UM-solution, consisting of 2% SDS, 8M urea, 5% 2-mercaptoethanol and 20 mM Tris HCl (pH= 7.5) following Konno & Imamura [8]. The final protein concentration was 20 mg/mL. The mixture was gently shaken overnight at room temperature to dissolve the sample. The protein profile of the different gels was monitored using SDS-PAGE analysis, 7.5 % acrylamide (Biorad, California, USA). The ChemiDoc XRS+ system controlled by Image Lab™ 5.1 (Bio Rad, California, USA) was used for processing of the SDS-page images.

**Colour of bean protein isolate gels**

Colour was determined on BPI gels from lots A and B with 14% and 17% BPI and 0% NaCl (G14-A, G17-A, G14-B, G17-B) using a portable colorimeter (Minolta Chroma MeterCr-200, Japan). The CIE Lab values were as originally defined by the Commission Internationale de l'Eclairage; L* (lightness), a* (redness) and b* (yellowness) of samples were evaluated on each BPI gel as described by Shevkani et al. (2014) [8]. Whiteness index (WI) of these isolates was also calculated using the equation WI=L*-3b* [8]. Six determinations were performed for each sample.

**Dynamic rheometry of bean protein isolate gels**

Small amplitude oscillatory shear (SAOS) tests were performed using a RS600 Haake-rheometer (Thermo Electron Karlsruhe, GmbH). The measurements were carried out using parallel plate (20 mm diameter and 1 mm gap). Lots A and B of BPI gels with better mechanical properties from puncture tests (14%, and 17% BPI concentration and 0% NaCl) were subjected to SAOS tests. These cylindrical gels (G14-A, G14-B, G17-A, G17-B) 20 mm in diameter were cut into disk-shaped slices around 1 mm thick. Samples rested for 15 min before analysis to ensure both thermal and mechanical equilibrium at the time of measurement .They were covered with a steel solvent trap to maintain the moisture content during testing. For all tests, temperature was controlled by a Peltier element in the lower plate and was kept at 20.0±0.1 °C. All the viscoelastic determinations were carried out in quintuplicate.

*Stress sweep tests*

Stress sweeps were performed at increasing stress values, at constant angular frequency (ω=6.28 rad/s). To determine the linear viscoelastic (LVE) region, amplitude sweeps were conducted by increasing the stress (*σ*) from 30 to 3000 Pa, and 300 points in the continuous mode were recorded. Changes in the strain (*γ*), storage modulus (*G’*), loss modulus (*G’’*), and complex modulus, *G**= (*G’*^2^+*G’’*^2^)^1/2^ were recorded. The limit parameters of the LVE range (*σ*_max_ and *γ_max_*) were obtained using *G** with a range of tolerable deviation of ± 10% from the initial value [8].

*Mechanical spectra*

Frequency sweep shows the frequency dependence of *G’* and *G’’* at fixed *γ* in the LVE range. In this test, a sinusoidal stress at fixed strain (*γ*=0.5%) was imposed on gels to ensure that the resulting *σ* in the sample would always fall in the LVE range. The viscoelastic parameters were determined over a range of ω values between 0.628 rad/s and 62.8 rad/s at 20 °C.

**Bioactive compounds of the bean protein isolate gels**

Different bioactive compounds (inositol phosphates, trypsin inhibitors, lectins, and α-galactosides) were analysed in the BPI and the selected BPI gels from lot B with 14 % and 17 % BPI and 0% NaCl (G14-B and G17-B). Inositol phosphates were analysed according to Burbano et al. [9] using a HPLC (Beckman System Gold Instrument, Los Angeles, CA, USA) and a PRP-1 column (150 x 4.1 mm i.d., 5 μm, Hamilton, Reno, Nevada, USA). Individual inositol phosphates (IP3-IP6) were quantified using an external standard (Sodium phytate; Sigma-Aldrich, St. Louis, MO, USA). The lectin content in the BPI gels was determined using an competitive indirect ELISA (enzyme-linked immunosorbent assay) according to the method described by Grant [10]. The PHA content was calculated using a calibration curve (0.001-1000 μg/mL) of pure PHA standard. *P. vulgaris* cvs Processor and Pinto were analysed in each assay as positive and negative controls, respectively. Trypsin inhibitors were obtained and determined as described by Pedrosa et al. [11] and trypsin inhibitor units (TIU) were determined using α-N-benzoyl-DL-arginine-p-nitroanilidehydrochloride (Sigma-Aldrich, St. Louis, MO, USA) as the trypsin substrate. One trypsin unit (TIU) was defined as that which gave a reduction in absorbance units at 410 nm of 0.01 relative to trypsin control reactions, using a 10 mL assay volume. α-Galactosides were analysed according to Pedrosa et al. [11] using a HPLC (Beckman System Gold instrument, Los Angeles, CA, USA) equipped with a refractive index detector, and a Spherisorb-5-NH2 column (250 x 4.6 mm i.d., Waters, Milford, MA, USA). The individual sugars were quantified by external standards (Sigma-Aldrich, St. Louis, MO, USA).

**References**

1. Pedrosa MM, Varela A, Domínguez-Timón F, Tovar CA, Moreno HM, Borderías AJ, Díaz MT (2020) Comparison of Bioactive Compounds Content and Techno-Functional Properties of Pea and Bean Flours and their Protein Isolates. Plant Foods Hum Nutr 75 (4):642-650. http://doi.org/10.1007/s11130-020-00866-4

2. Bradford MM (1976) A rapid and sensitive method for the quantitation of microgram quantities of protein utilizing the principle of protein-dye binding. Anal Biochem 72:248-254

3. Cuadrado C, Guillamon E, Goyoaga C, Pedrosa MM, Altares P, Burbano C, Muzquiz M, Romero C (2004) Modifications of seed storage proteins during germination and seedling growth of faba bean cotyledons. Recent Advances of Research in Antinutritional Factors in Legume Seeds and Oilseeds. Wageningen Academic Toledo (Spain)

4. AOAC (2000) Official Methods of Analysis. Association of Official Analytical Chemists, (16th ed.) edn. Association of Official Analytical Chemists. AOAC International, Washington, DC

5. Chau CF, Cheung PCK (1998) Functional properties of flours prepared from three Chinese indigenous legume seeds. Food Chem 61 (4):429-433. <http://doi.org/10.1016/S0308-8146(97)00091-5>

6. Moreno HM, Domínguez-Timón F, Díaz MT, Pedrosa MM, Borderías AJ, Tovar CA (2020) Evaluation of gels made with different commercial pea protein isolate: Rheological, structural and functional properties. Food Hydrocoll 99:105375. <https://doi.org/10.1016/j.foodhyd.2019.105375>

7. Shand PJ, Ya H, Pietrasik Z, Wanasundara PKJPD (2007) Physicochemical and textural properties of heat-induced pea protein isolate gels. Food Chem 102 (4):1119-1130. <https://doi.org/10.1016/j.foodchem.2006.06.060>

8. Konno K, Imamura K (2000) Identification of the 150 and 70 kDa Fragments Generated during the Incubation of Salted Surimi Paste of Walleye Pollack. Nippon Suisan Gakkaishi (Japanese Edition) 66:869-875. http://doi.org/10.2331/suisan.66.869

9. Burbano C, Muzquiz M, Ayet G, Cuadrado C, Pedrosa MM (1999) Evaluation of antinutritional factors of selected varieties of Phaseolus vulgaris. J Sci Food Agric 79:1468-1472. <https://doi.org/10.1002/(sici)1097-0010(199908)79:11><1468::aid-jsfa387>3.0.co;2-g

10. Grant G (1991) Lectins. In: D'Mello, JPF, Duffus, CM, Duffus, JH (Eds), Toxic Substances in Crop Plants. Woodhead Publishing. Cambridge, England, <http://dx.doi.org/10.1533/9781845698454.49>

11. Pedrosa MM, Cuadrado C, Burbano C, Muzquiz M, Cabellos B, Olmedilla-Alonso B, Asensio-Vegas C (2015) Effects of industrial canning on the proximate composition, bioactive compounds contents and nutritional profile of two Spanish common dry beans (*Phaseolus vulgaris* L.). Food Chem 166:68-75. <https://doi.org/10.1016/j.foodchem.2014.05.158>
